# LXXLL Peptide Converts Transportan 10 to a Potent Inducer of Apoptosis in Breast Cancer Cells

**DOI:** 10.3390/ijms15045680

**Published:** 2014-04-03

**Authors:** Kairit Tints, Madis Prink, Toomas Neuman, Kaia Palm

**Affiliations:** 1 Protobios LLC, Mäealuse 4, Tallinn 12618, Estonia; E-Mails: madis@protobios.com (M.P.); tom@protobios.com (T.N.); 2 Competence Centre for Cancer Research, Akadeemia tee 15, Tallinn 12618, Estonia; 3 Department of Gene Technology, Tallinn University of Technology, Akadeemia tee 15, Tallinn 12618, Estonia

**Keywords:** SRC-1, LXXLL, cancer, cell penetrating peptide

## Abstract

Degenerate expression of transcription coregulator proteins is observed in most human cancers. Therefore, in targeted anti-cancer therapy development, intervention at the level of cancer-specific transcription is of high interest. The steroid receptor coactivator-1 (SRC-1) is highly expressed in breast, endometrial, and prostate cancer. It is present in various transcription complexes, including those containing nuclear hormone receptors. We examined the effects of a peptide that contains the LXXLL-motif of the human SRC-1 nuclear receptor box 1 linked to the cell-penetrating transportan 10 (TP10), hereafter referred to as TP10-SRC1_LXXLL_, on proliferation and estrogen-mediated transcription of breast cancer cells *in vitro*. Our data show that TP10-SRC1_LXXLL_ induced dose-dependent cell death of breast cancer cells, and that this effect was not affected by estrogen receptor (ER) status. Surprisingly TP10-SRC1_LXXLL_ severely reduced the viability and proliferation of hormone-unresponsive breast cancer MDA-MB-231 cells. In addition, the regulation of the endogenous ERα direct target gene *pS2* was not affected by TP10-SRC1_LXXLL_ in estrogen-stimulated MCF-7 cells. Dermal fibroblasts were similarly affected by treatment with higher concentrations of TP10-SRC1_LXXLL_ and this effect was significantly delayed. These results suggest that the TP10-SRC1_LXXLL_ peptide may be an effective drug candidate in the treatment of cancers with minimal therapeutic options, for example ER-negative tumors.

## 1. Introduction

Breast cancer is the most common invasive cancer in women worldwide and numerous aggressive and innovative therapies have been developed that target steroid hormone signaling. However, many women do not benefit from these treatments, since 25%–50% of hormone-receptor-positive breast cancers are *de novo* resistant to endocrine therapy, and most, if not all, metastatic breast cancers develop resistance [1]. The interaction specificity of a short linear LXXLL-motif with nuclear hormone receptors is well described [2–4] and in this study we examined their potential role as anti-cancer therapeutics in breast cancer treatment. We hypothesized that disrupting transcription factor function using peptides carrying a short LXXLL-motif may desensitize cells to nuclear hormones and have a cytotoxic effect. This may provide a novel approach to developing bioactive cell-penetrating peptides (bioportides) as chemotherapeutic agents.

Coregulator proteins facilitate interactions of transcription factors with the general transcriptional machinery and elicit efficient transcriptional activation of multiple target genes [5]. The p160 steroid receptor coactivator (SRC) family contains structurally highly conserved proteins, including SRC-1 (NCoA-1), SRC-2 (TIF2/GRIP-1/NCoA-2), and SRC-3 (ACTR/AIB1/RAC3/SRC-3/TRAM-1) [6,7], with overlapping functions in regulating nuclear receptor (NR) signaling [8]. NR coactivators do not directly bind DNA, but interact with ligand-bound NRs to recruit other components of a large coactivator complex to the hormone response elements of a target gene. The central region of the p160 SRC proteins contains a nuclear receptor interaction domain consisting of three short alpha-helical recognition motifs with LXXLL sequences, which are responsible for direct association of the coactivator with a specific NR [2,3,9]. LXXLL motifs are defined as leucine rich amphipathic helices with limited leucine substitution for hydrophobic residues and at least one negatively charged amino acid in an X position. Furthermore, functional LXXLL motifs occur in proteins that do not directly interact with NRs, including the transcription factors c-Myb [10], STAT-6 [11], CREB and p300 [7], and mediator subunits [12,13].

NRs regulated by SRC-1 include the progesterone receptor (PR), glucocorticoid receptor (GR), estrogen receptor alpha (ERα), thyroid receptor (TR), retinoid X receptor (RXR), hepatocyte nuclear factor 4 (HNF4α), and peroxisome proliferator-activated receptor γ (PPARγ) [8,14,15]. The binding affinity of SRC-1 for NRs depends on the respective domain of interaction. The central domain of SRC-1 has high affinity for ER, vitamin D receptor (VDR), retinoic acid receptor (RAR), and TR [16], but it is unable to bind the androgen receptor (AR) and exhibits a poor affinity of binding for GR. Förster resonance energy transfer (FRET) data demonstrated that the complex formed between ERα and SRC-1 exhibits a particularly high binding affinity, as compared to other SRC-1/NR complexes [17]. SRC-1 is also capable of coactivating non-steroidal transcription factors, such as AP-1, SRF, NFκβ, human Ets2, and HOXC11 [18–23], and can promote gene transcription by interacting with kinases, phosphatases, ubiquitin and small ubiquitin-related modifier ligases, histone acetyltransferases, and histone methyltransferases [24]. Subsequently, SRC-1 regulates many diverse physiological functions with numerous molecular targets including genes involved in cell cycle control and energy metabolism pathways, such as glycolysis, glycogen synthesis, and fatty acid synthesis [25–27].

Recent work has indicated that the SRC genes are subject to amplification and over-expression in different human cancers, in particular in steroid hormone-promoted breast and prostate cancers [28–31]. The molecular mechanisms by which SRCs promote breast and prostate cancer cell proliferation and survival have actively been investigated, and the specific contributions of SRC-1 in tumor cell migration, invasion, and metastasis have been examined using various cell [32] and genetically manipulated mouse models [26,33–35]. These studies have identified new challenges for targeting SRC-1 in cancer research and therapy.

Extensive studies have shown that the LXXLL sequence is necessary and sufficient for the binding of p160 SRC proteins to NRs and for stimulation of transcriptional activity. For example, in MCF-7 breast cancer cells, SRC-1 over-expression potentiates cell growth that is further stimulated by estrogen action in accordance with the increased expression of estrogen-responsive genes [32]. LXXLL-like motifs mediate transcription factor-coactivator interactions in a variety of complexes. These short, linear motifs bind their cognate receptors with low to moderate affinity, which is ideal for transient signaling interactions [36]. In the search for inhibitors, various short peptide derivatives based on the LXXLL sequence have been demonstrated to disrupt the interactions of coactivators with NRs [37–39]. There are also a few reports on non-peptide inhibitors designed to bind NRs and block the binding of coactivators [40–45]. Recently reported analysis of nuclear receptor coregulator motifs revealed that 149 out of 303 coregulators have at least one LXXLL motif (Nuclear Receptor Signaling Atlas, http://www.nursa.org), indicating that it is a common but not universal motif for coregulators.

Previous studies have indicated that cell-penetrating peptides (CPPs) are highly efficient intracellular delivery vehicles of bioactive molecules, including peptides, proteins, oligonucleotides, and other bio-conjugates [46]. CPPs are short, cationic and/or amphipathic peptides, usually <30 amino acids in length, that efficiently translocate into cells [47]. The majority of studies employ sychnologically organized CPPs that combine an active cargo (message) with an inert CPP (address) as a tandem linkage [48]. Since it has been suggested that peptide analogues mimicking short linear motifs, such as LXXLL, may disrupt SRC function [38], we constructed a sychnologically organized TP10-SRC1_LXXLL_ peptide based on the SV40 nuclear localization signal (NLS) chimeric CPP that is an analogue of mastoparan extended at the amino terminus with a sequence derived from galanin, TP10 [49], and a sequence containing the NR box 1 of human SRC-1 [50]. The cell penetrating properties of TP10 [49] and hepta-arginin (R_7_) [51], and their capacity to carry molecular cargoes into cells have been well established. In this study, TP10, as an effective vehicle for the delivery of biologically active peptide cargos with a relatively low intrinsic toxicity, was selected to deliver the SRC1_LXXLL_ peptide to the cellular targets.

Our data reveal that the bioportide TP10-SRC1_LXXLL_ displays severe cytotoxicity in breast cancer cells, which we propose is not associated with the disruption of ER function.

## 2. Results and Discussion

### 2.1. Results

To generate bioportide peptides (Table 1) that could target the activity of nuclear hormone receptors and potentially inhibit proliferative disorders like breast cancer, we selected a short linear LXXLL-motif sequence that is present in SRC-1 NR box 1 and is reported to have a high affinity for ER [2]. To deliver these motif-carrying peptides to cells, two different CPPs, namely TP10 and R7 were used. The designed bioportides were *N*-terminally extended with the SV40 NLS to direct nuclear localization. As a negative control, we used peptides in which the NR box 1 sequence was substituted with a randomly chosen non-structured fragment of SRC-1, encompassing amino acids 1222–1245.

#### 2.1.1. Cellular Toxicity of TP10-SRC1_LXXLL_ Peptide

**Figure 1.**
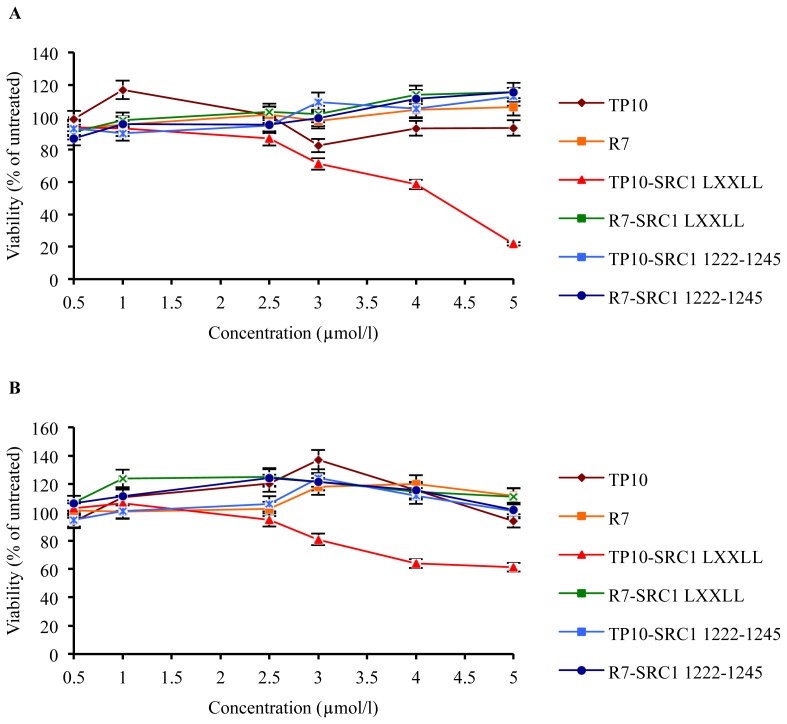
Effects of SRC-1 bioportides on cancer and primary fibroblast cells. (**A**) Viability of MCF-7 cells; (**B**) Viability of FB188 cells. Cells were seeded in 96-well plates at a density of 3000 or 2000 cells/well, respectively, and treated after every 24 h with the indicated peptide concentrations. Viability of both cell types was monitored at day 3 via the WST-1 assay. The values shown represent the mean values of one experiment, carried out in triplicate (mean ± SD). In total, three independent experiments were performed.

As cell death can occur at high concentrations of TP10 [47] and polyarginine [52], it was necessary to assess the cellular toxicity of the bioportides and respective controls. The viability of MCF-7 breast cancer cells and primary fibroblasts was tested using the WST-1 assay over a period of 3 days using increasing concentration of peptides (Figure 1). Fibroblasts are the most common type of the healthy cells in the stroma surrounding the breast cancerous tumor cells. Therefore, we specifically investigated how the bioportides affect proliferation and viability of the normal cells that physiologically occur in the vicinity of cancer cells. These studies showed that all peptides at concentrations of 0.5, 1, and 2.5 μM, and TP10 and R_7_ even at concentrations of 5 μM did not have any effect on the viability of MCF-7 breast cancer cells and human primary fibroblasts. However, at concentrations of 5 μM, TP10-SRC1_LXXLL_ was significantly more toxic for MCF-7 cells than all other peptides. At concentrations equal or higher than 4 μM, TP10-SRC1_LXXLL_ affected the viability of primary fibroblasts, whereas control peptides did not have any effect (Figure 1A,B). Apart from TP10-SRC1_LXXLL_, all other peptides, including TP10-SRC1_1222–1245_, R7-SRC1_1222–1245_, and R7-SRC1_LXXLL_ were not toxic to other cells analyzed, such as HeLa cervical cancer cells (data not shown). These data indicate that TP10-SRC1_LXXLL_ is specifically cytotoxic, that is, the inclusion of the LXXLL of NR box 1 alone is necessary but insufficient without the presence of the TP10 sequence for the observed cytotoxicity.

Differential cellular sensitivity to TP10-SRC1_LXXLL_ peptide action may be related to different mechanisms of uptake of cargo-loaded TP10 in different cell types [49,53]. As recently summarized [54], CPP vectors can often display unwanted activities by virtue of their abilities to interfere with a variety of cellular processes. Particularly at higher concentrations (>10 μM), some CPPs can adversely modify membrane integrity and compromise cellular viability [55]. Other studies strongly suggest that the activity of a bioactive cargo can profoundly be influenced by the CPP to which it is sychnologically conjugated [56,57]. Given that tumor cells have more acidic extracellular environments than normal tissues, and cell membranes of cancer cells have higher negative charges than normal cells [58], it is conceivable that cancer cells provide a unique cellular environment that is favorable for TP10-SRC1_LXXLL_ peptide internalization and intracellular delivery.

#### 2.1.2. Bioportide TP10-SRC1_LXXLL_ Translocates to the Nucleus of MCF-7 Cells

**Figure 2.**
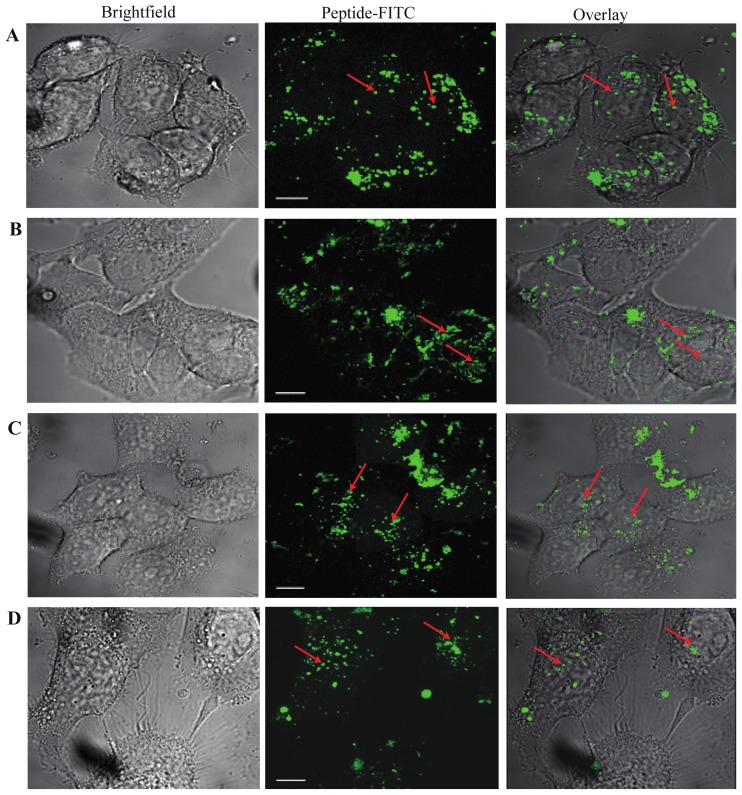
Cellular distribution of SRC-1 bioportides. (**A**) TP10-SRC1_LXXLL_; (**B**) TP10-SRC1_1222–1245_; (**C**) R_7_-SRC1_LXXLL_; (**D**) R_7_-SRC1_1222–1245_. Confocal microscopy of MCF-7 cells exposed to 5 μM FITC-labeled peptides for 1 h. The cells were washed 3 times with stimulation medium before confocal microscopy of live cells was carried out. Nuclear localization of peptides is indicated with red arrows (63× magnification; scale bar 10 μm).

To further examine the cellular penetration potential of the CPP-conjugated SRC-1 derived peptide sequences, confocal immunofluorescence analysis was performed with live cells to discriminate between extra- and intracellular localization of the peptides and to identify their subcellular structural localization. As shown in Figure 2, at 1 h, all analyzed peptides, including TP10-SRC1_LXXLL_, TP10-SRC1_1222–1245_, R7-SRC1_LXXLL_, and R7-SRC1_1222–1245_, were detected in the cytoplasm and nucleus of MCF-7 cells. While most of the TP10-SRC1_LXXLL_ signal was seen in the cytoplasm, nuclear fluorescence was evident.

Conjugation of bioportides to nuclear targeting agents, including small molecules and proteins (apoptin) has been demonstrated to be an approach that is feasible of expanding the CPP-based therapeutic arsenal to target transcription [59]. Though there remains a lack of consensus, it is obvious and confirmed by our study that cargo peptides delivered by CPP technologies are able to escape endosomal entrapment and target specific intracellular organelles and compartments, such as the cellular nucleus. On the other hand, NRs as potential targets of SRC1_LXXLL_ may localize within several sub-cellular compartments. Therefore this extra-nuclear fluorescence could represent the ability of TP10-SRC1_LXXLL_ to bind to cytoplasmic NR.

#### 2.1.3. TP10-SRC1_LXXLL_ Affects Viability of Breast Cancer Cells Independently of ER-Mediated Transcription

In order to elucidate whether the bioportides TP10-SRC1_LXXLL_ and TP10-SRC1_1222–1245_ could interfere with ER-mediated cell signaling, MCF-7 and MDA-MB-231 breast cancer cells were treated with respective peptides at 5 μM concentration under estrogen-stimulated and insulin deprived conditions every 24 h for a 4-day period. In this study, MCF-7 cells were used as ER-positive and MDA-MB-231 as ER-negative breast cancer cell prototypes. This choice was made based on the fact that MCF-7 cells, being ER-responsive, are absolutely dependent on estrogen and exogenous growth factors for proliferation and become quiescent in media supplemented with growth factor-inactivated serum [60], whereas the growth of MDA-MB-231 cells is independent of estrogen action.

**Figure 3.**
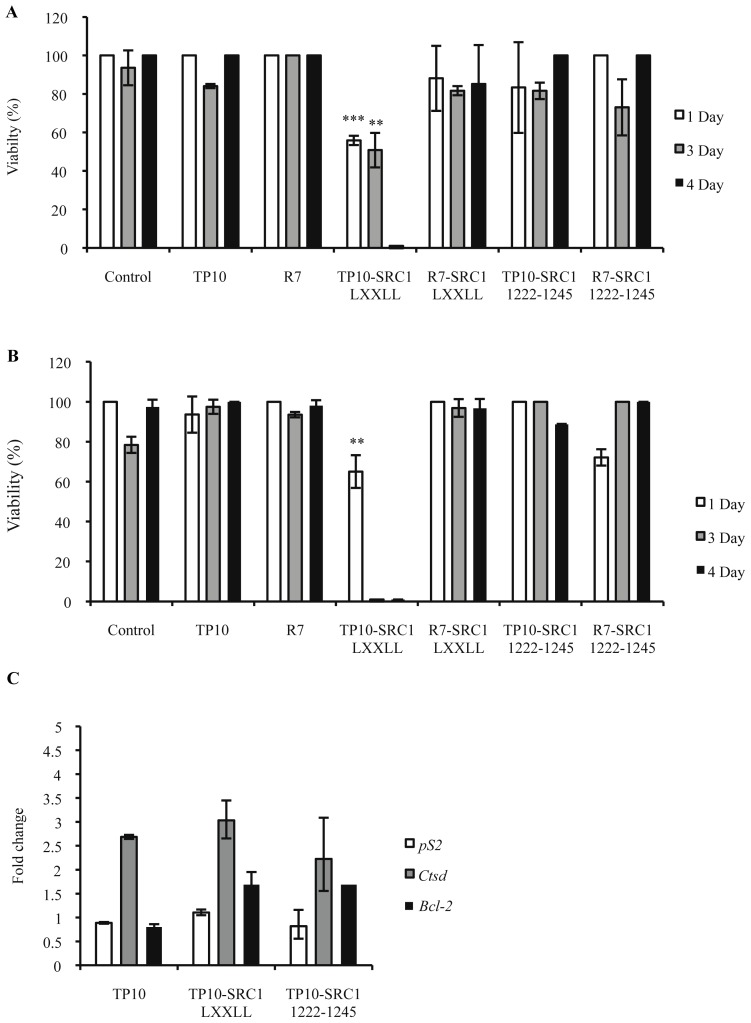
Inhibition of cell viability by SRC-1 bioportides and their influence on ERα-mediated gene transactivation. (**A**) MCF-7 cells; (**B**) MDA-MB-231 cells. Cells were seeded in 24-well plates at low density and were treated every 24 h for 4 days with 5 μM peptide. Cell proliferation was measured after 1, 3, and 4 days of peptide treatment via a propidium iodide viability assay. Differences were found to be statistically significant with ** *p* < 0.01, *** *p* < 0.001; (**C**) Endogenous ERα target genes *pS2*, *CTSD*, and *BCL2* mRNA expression analysis. MCF-7 cells were cultured with 5 μM of the indicated peptides and analyzed on day 2 of treatment. The fold change is the value compared with untreated cells. The values shown represent the mean values of one representative experiment, carried out in triplicate (mean ± SD). In total, three independent experiments were performed.

Propidium iodide (PI) staining-based cell viability assays were carried out and the TP10-SRC1_LXXLL_ peptide was found to have significant cytotoxic effects on MCF-7 and MDA-MB-231 cells in contrast to R_7_-SRC1_LXXLL_, TP10-SRC1_1222–1245_ and R_7_-SRC1_1222–1245_, which did not affect survival compared with untreated cells or cells treated with control peptides (Figure 3). TP10-SRC1_LXXLL_ was found to have a significant effect on survival of MCF-7 and MDA-MB-231 cells as soon as day 1 (Figure 3A,B). Data analysis at day 3 showed that exposure of MCF-7 and MDA-MB-231 cells to TP10-SRC1_LXXLL_ (5 μM) killed 50% and 99% cells, respectively. By day 4, the number of viable cells in both cases was less than 1%. These data indicate that TP10-SRC1_LXXLL_ displays differential cytotoxic effects that affect breast cancer cell survival.

Expression of *pS2*, a gene regulated by estrogen, was analyzed in MCF-7 cells using quantitative RT-PCR to assess whether TP10-SRC1_LXXLL_ affects survival through the ER-signaling pathways. No down-regulation of *pS2* mRNA levels was observed in MCF-7 cells at day 2 upon treatment with TP10-SRC1_LXXLL_, TP10-SRC1_1222–1245_, or with TP10 (Figure 3C). Furthermore, *pS2* expression was not affected in MDA-MD-231 cells upon treatment with either of the peptides. Expression of other estrogen responsive marker genes, *i.e*., cathepsin D (*CTSD*) and B-cell lymphoma 2 (*BCL2*), were slightly elevated, but similar to TP10 or TP10-SRC1_1222–1245_ treated cells (Figure 3C). Together, these results indicated that TP10-SRC1_LXXLL_ did not interfere with ER-regulated transcriptional activation of *pS2*.

#### 2.1.4. Effect of TP10-SRC1_LXXLL_ on Apoptosis

**Figure 4.**
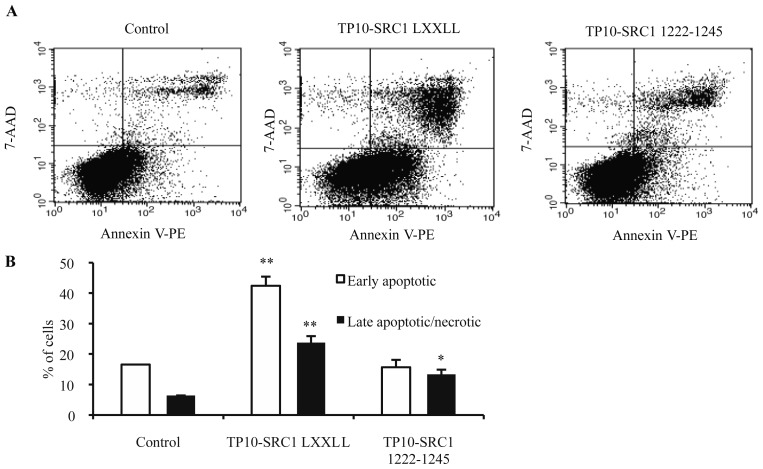
Flow cytometric detection of apoptosis by annexin V and 7-amino-actinomycin D (7-AAD) staining. (**A**) Representative dot plots of 7-AAD versus annexin-V-PE. MCF-7 cells were left untreated (control) or were treated with 5 μM of peptide for 24 h in media supplemented with 1% serum. The lower left quadrant contains the vital (double negative) population; the lower right contains the apoptotic (annexin V^+^/7-AAD^−^) population; the upper right contains the late apoptotic/necrotic (annexin V^+^/7-AAD^+^) population; and the upper left contains the pre-necrotic (annexin V^−^/7-AAD^+^) population; (**B**) Average percentage of apoptotic and late apoptotic/necrotic cells counted from dot plots taken from one representative experiment, error bars indicate mean ± SD. Data are statistically significant with * *p* < 0.05, ** *p* < 0.01. In total, three independent experiments were performed. 7-AAD, 7-amino-actinomycin D; PE, phycoerythrin; TP10-SRC1_LXXLL_, sequence of SRC1 NR box 1 with *N*-terminal TP10; TP10-SRC1_1222–1245_, sequence of randomly chosen non-structured fragment of SRC1 with *N*-terminal TP10.

To assess the mechanism of MCF-7 cell death upon TP10-SRC1_LXXLL_ treatment, cytofluorimetric PE-annexin-V analysis was used. Representative dot plots of annexin V/7-AAD staining analyses are shown in Figure 4. There was no significant difference in the amount of annexin V (+)/7-AAD (−) stained MCF-7 cells upon 24 h of using TP10-SRC1_1222–1245_ peptide (5 μM) treatment compared with untreated cells (control). In contrast, treatment with TP10-SRC1_LXXLL_ (5 μM) at 24 h yielded a predominant population of annexin V positive and 7-AAD negative cells that were considered as early apoptotic, whilst a smaller population of cells that stained for both 7-AAD and annexin V had apparently died of necrosis (Figure 4). Taken together, these data suggested that apoptosis, rather than necrosis, was the main mode of action of TP10-SRC1_LXXLL_ in breast cancer cells.

### 2.2. Discussion

Data presented in this report are the first to demonstrate that the LXXLL motif in combination with TP10 can induce cancer cell death. Using different peptide concentrations, we established that at a concentration of 5 μM, TP10-SRC1_LXXLL_ is highly cytotoxic to carcinoma MCF-7, MDA-MB-231, and HeLa cells. Our data show that the LXXLL motif of SRC-1 contributes to cytotoxicity observed in TP10-SRC1_LXXLL_ treated carcinoma cells, since TP10 alone does not induce apoptosis at similar concentrations. However, our data also suggest that the activity of SRC1_LXXLL_ is profoundly influenced by the TP10 peptide to which it is sychnologically conjugated. Additionally, we provide evidence that different cancer cells show significantly increased sensitivity to TP10-SRC1_LXXLL_ (≤5 μM) compared to human fibroblasts, which leads us to conclude that TP10-SRC1_LXXLL_ is highly cytotoxic to carcinoma cells.

Several studies have shown that peptido-mimetics can act as selective, high-affinity inhibitors of coactivator binding to NRs [38,39], supporting the idea of targeting this interface by the design of effective peptide-based drug candidates. Supported by our localization data, we investigated the purported role of TP10-SRC1_LXXLL_ as a potential inhibitor of SRC-1-ER interactions and analyzed its effects on ER-positive breast cancer cell survival. Surprisingly, we determined that LXXLL NR box 1-containing bioportides affect the survival of both estrogen-responsive and estrogen-unresponsive breast cancer cells, although it appears that TP10-SRC1_LXXLL_ peptides have a slightly more profound effect on the survival of the estrogen-unresponsive cells (Figure 3B). This could be attributed to the potential of TP10-SRC1_LXXLL_ compounds to interfere with the activity of other signaling pathways that are crucial to breast cancer cell survival, independently of estrogen signaling pathways. It is well-known that during breast cancer progression, many initially ER-positive tumors lose ER expression and attain hormone unresponsiveness [61,62]. Regarding the potential molecular mechanisms behind TP10-SRC1_LXXLL_’s role as a cell death inducer, both in estrogen responsive and unresponsive breast cancer cells, we assumed different possible scenarios. First, the activity conveyed through LXXLL motifs is not limited to nuclear-receptor signaling given that these motifs participate in various protein-protein interactions associated with different aspects of transcriptional regulation. For example E-proteins, the family of transcription factors involved in the regulation of cell growth, differentiation, and apoptosis [63–67], interact with CBP/p300 via LXXLL motifs. In E-protein transactivation, CBP/p300 is recruited by an LXXLL motif in the *N*-terminal activation domain 1 (AD1); a mechanism that is similar to nuclear receptor signaling. It is well established that signaling through LXXLL recruits coactivators or corepressors and controls basal transcription by promoting changes in histone and chromatin formation. These interactions are mediated by short peptide motifs [68,69]. So, one may speculate on a role for HLH proteins in recruiting NCoa1 in breast cancer cells and inducing cell death. Alternatively, the TP10-SRC1_LXXLL_-induced cell death might be regulated through the activity of the transcription Mediator complex. It has been shown that the LXXLL motifs of MED1 are required for nuclear receptor-mediated transcription *in vitro*. Provided that Med1 is differently expressed in mammary epithelial cells suggests a role of MED1 LXXLL motifs in ERα-mediated functions of early mammary gland development, since mammary ductal growth and branch morphogenesis are significantly impaired in Med1*^KI^*^/^*^KI^* mice relative to wild-type mice [70]. Consistent with the observation, Med1*^KI^*^/^*^KI^* mammary epithelial cells show a significant reduction in the expression of a number of known ERα target genes, including *pS2*. Interestingly and in good support of our data, the expression of these genes is no longer responsive to estrogen stimulation in isolated mammary epithelial cells. Furthermore, estrogen-stimulated mammary duct growth is blocked by MED1 LXXLL mutations *in vivo*. Finally, the bHLH–PAS domains of NCOAs bind to many transcription factors, including signal transducer and activator of transcription 6 (STAT6) and p53, and to other co-activators, including COCOA (also known as CALCOCO1), friend leukaemia integration 1 (FLI1), and BRG1-associated factor 57 (BAF57; also known as SMARCE1) (see references in Bersten *et al.* [71]). It is quite obvious that interaction through the LXXLL domain of NCOA1 interferes or acts in a synergistic manner with any of these transcription factors and contributes to cancer-specific cell death.

Furthermore, we report here that this bioportide has no impact on ER-mediated mechanisms in breast cancer cells, provided that three genes known to be ER-responsive in MCF-7 cells [32,72] were not modulated at the level of transcription upon TP10-SRC1_LXXLL_ treatment. Therefore, it was concluded that TP10-SRC1_LXXLL_ is not affecting nuclear hormone-induced transcription. However, TP10-SRC1_LXXLL_ clearly acted as a potent pro-apoptotic agent on breast cancer cells. Recently, a structurally similar peptide D-NuBCP-9-r8 with a FSRSLHSLL sequence targeting *BCL2*, in synergy with the cell penetrating octa-arginine (R_8_) sequence caused rapid membrane blebbing leading to cell necrosis independent of *BCL2* expression at high (>10 μM) concentrations [73]. It is reasonable to suggest, based on our findings, that TP10-SRC1_LXXLL_ could bind a spectrum of intracellular protein targets, although at present we cannot exclude the possibility that some of its activities may result from TP10-SRC1_LXXLL_ directly occupying ERα binding pockets, provided that TP10-SRC1_LXXLL_ has the potential to colocalize with ERα in the nucleus of MCF-7 cells.

Earlier findings described the use of LXXLL-containing peptides in targeting nuclear receptors [74,75], ATF-2 derived peptides to influence the TGF-β pathway [76], and peptides interfering with the DNA binding activity of the transcription factor E2F [77]. While the specific mechanisms underlying sensitivity to TP10-SRC1_LXXLL_ inhibition remain to be defined, it is clear from the results of our studies that the apoptotic activity of TP10-SRC1_LXXLL_ is not the consequence of sole inhibition of ER-transcriptional activity and that LXXLL derived of SRC-1 is interfering with the fundamental processes of survival of carcinoma cells. Despite the identified importance of SRC1_LXXLL_ carrying bioportides on ER-dependent transcriptional activity, our studies also underscore the role of TP10 in contributing to the apoptotic effects of SRC1_LXXLL_ in the moiety of TP10-SRC1_LXXLL_ in carcinoma cells.

The therapeutic potential of SRC1_LXXLL_-containing bioportides in life-threatening diseases has made them subject of intense pharmacological research for NR-dependent health conditions [37–39]. As also exemplified by our studies, bioportides carrying SRC1_LXXLL_ display potent and therapeutically beneficial activities, both in a NR-dependent and independent manner, that could be further improved by adding, for example, a homing peptide sequence. We predict that targeting other complexes that SRC1_LXXLL_ motif-carrying bioportides can associate with, such as Mediator, may allow the development of additional ways for the selective induction of hormone-independent cancer cell death. Of course, transcription complex targeting compounds might carry the risk of some toxicity, but the benefit of stopping overactive, cancer-specific transcription should outweigh the risk.

## 3. Experimental Section

### 3.1. Peptide Synthesis, Purification and Analysis

All peptides were purchased from GL Biochem (Shanghai, China), JPT (Berlin, Germany), or Celecure (Tallinn, Estonia); purity > 95%. For fluorescence microscopy (Eclipse 80i, Nikon, Melville, NY, USA) and confocal live cell imaging, fluorescein isothiocyanate (FITC, Thermo Scientific, Rockford, IL, USA) labeled peptides were used. The predicted masses of all peptides (average M+ H+) were confirmed with an accuracy of +1 by Matrix-assisted laser desorption/ionization-time of flight (MALDI-TOF) mass spectrometry using Autoflex II TOF/TOF (Burker Daltonics, Bremen, Germany) and flexControl 3.0 software (Burker Daltonics, Bremen, Germany).

### 3.2. Cell Culture

MCF-7 and MDA-MB-231 cells were obtained from the American Type Culture Collection (ATCC, Boras, Sweden). Primary human fibroblasts were obtained, with approval of the local ethical committee (TMEK No. 2551), from skin biopsies using an in-house migration assay. MCF-7 cells were routinely maintained in EMEM (GIBCO, Grand Island, NY, USA), supplemented with 10% FBS (PAA, Pasching, Austria) and 10 μg/mL human insulin (Sigma, St. Louis, MO, USA). MDA-MB-231 and primary human fibroblasts were maintained in DMEM with high glycose (PAA), supplemented with 10% FBS (PAA). All media contained l-glutamine (0.1 mg/mL) (PAA) and were supplemented with 1% penicillin/streptomycin (PAA). Cells were cultured in a humidified atmosphere of 5% CO_2_ at 37 °C.

### 3.3. Viability Assay

Cytotoxicity of peptides was measured via the WST-1 conversion assay (Roche, Mannheim, Germany). FB188 and MCF-7 cells were seeded in 96-well plates at a density of 2000 or 3000 cells/well, respectively, and allowed to adhere overnight. Subsequently the cells were pre-incubated for two days in medium containing 1% FBS without supplementary insulin and exposed to the indicated concentrations of the respective peptide for up to 3 days. WST-1 conversion was determined by colorimetric analysis at 450 nm. Briefly, WST-1 reagent (1/10 of sample volume) was added directly to the growth medium and incubated at 37 °C for 4 h. The absorbance was determined using a microplate reader (Molecular Devices, Sunnyvale, CA, USA) at 450 nm. Untreated cells were designated a cell survival value of 100%.

### 3.4. Proliferation Assay

Cells were plated at low density in 24-well plates in standard growth medium, as described above. Before peptide treatment, MCF-7 cell growth was impeded by growing cells in 1% FBS containing medium without addition of insulin. At the day of treatment, peptides were added to the growth medium containing 1% FBS to the indicated final concentration. The procedure was repeated every 24 h for 4 days; samples were drawn after 1, 3, and 4 days of treatment and analyzed by cell counting using the propidium iodide viability assay (Nucleocounter, ChemoMetec, Allerød, Denmark) according to manufacturer’s instructions.

### 3.5. Quantitative RT-PCR

For quantitative RT-PCR (qPCR) analysis, MCF-7 cells were treated as described above for the proliferation assay. On day 2, cells were lysed and total RNA was extracted using a commercial RNAqueous Kit (Ambion, Austin, TX, USA). Genomic DNA was removed with DNase I treatment (Ambion) according to the supplier’s protocol. RNA was reverse-transcribed using SuperScript III Reverse Transcriptase (Invitrogen, Carlsbad, CA, USA). Subsequently, qPCR was performed using Platinum SYBR Green (Invitrogen) according to the manufacturer’s protocol. The ER target genes cDNA was amplified using the following primers: *pS2* forward primer 5′-CCCCTGGTGCTT CTATCCTA-3′, reverse primer 5′-GATCCCTGCAGAAGTGTCTAAAA-3′; *CTSD* forward primer 5′-CATCTTCTCCTTCTACCTGAGCA-3′, reverse primer 5′-GTCTGTGCCACCCAGCAT-3′; *BCL2* forward primer 5′-TTGACAGAGGATCATGCTGTACTT-3′, reverse primer 5′-ATCTTTATTTCATG AGGCACGTT-3′. Input levels were related to *GAPDH* housekeeping gene mRNA levels, using the forward primer 5′-CTCTCTGCTCCTCCTGTTCGAC-3′ and reverse primer 5′-TGAGCGATGTGGC TCGGCT-3′. Average values and SD were obtained from triplicate analyses. All primers were purchased from Microsynth (Balgach, Switzerland).

### 3.6. Confocal Microscopy

Cells were plated in 35 mm sterile glass base dishes (Greiner bio-one, Frickenhausen, Germany) and grown to subconfluency in medium containing 10% FBS and 1% penicillin/streptomycin in a humidified atmosphere with 5% CO_2_ at 37 °C. Before peptide treatment, the growth medium was gently removed and cells were washed once with pre-warmed stimulation medium (without phenol red and serum). Cells were incubated with 5 μM FITC-labeled peptides in stimulation medium for 1 h at 37 °C in a humidified atmosphere with 5% CO_2_, washed 3 times with stimulation medium, and analyzed using a LSM 510 Meta confocal microscope (Carl Zeiss, Jena, Germany).

### 3.7. Detection of Apoptosis with Annexin V and 7-Amino-Actinomycin D (7-AAD) Staining

Annexin V staining was measured using the PE-annexin-V Apoptosis Detection Kit I (BD Biosciences, San Diego, CA, USA). MCF-7 cells (3 × 10^5^) were treated for 24 h with 5 μM peptide in growth medium supplemented with 1% FBS and without insulin. Cells were washed twice with cold PBS and then resuspended in 1× Binding Buffer, 100 μL of the solution (1 × 10^5^ cells) was used to stain cells with 5 μL PE annexin V and 5 μL 7-AAD for 15 min at room temperature in the dark. Following the addition of 0.4 mL of 1× Binding Buffer, fluorescence of annexin V and 7-AAD was detected in the FL-2 and FL-3 channels, respectively, within 1 h using a FACS Calibur flowcytometer (BD Biosciences, Sparks, MD, USA). Unstained and untreated cells were used to establish the instrument settings. Statistical analyses were performed using CellQuest™ Pro software (BD Biosciences, Sparks, MD, USA).

### 3.8. Statistical Analysis

Statistical analysis was performed using an unpaired Student’s *t*-test with a 2-tailed *p* value. Differences were considered significant at *p* < 0.05.

## 4. Conclusions

Our data show that the bioportide TP10-SRC1_LXXLL_ displays severe cytotoxicity in breast cancer cells, ultimately triggering apoptosis. TP10-SRC1_LXXLL_ induced breast cancer cell death in a dose-dependent manner and this effect was not affected by estrogen receptor status given that TP10-SRC1_LXXLL_ severely reduced the viability and proliferation of hormone-unresponsive cells. This activity of TP10-SRC1_LXXLL_ as a potent inducer of apoptosis may be useful in cancer therapeutics, in particular for ER-negative tumors. There are no specific drugs for ER-negative breast cancer; therefore, we consider our early findings significant and our results might provide the basis for the scientific community to further develop therapeutics for targeting these types of cancers.

Anti-estrogen drugs are widely used for hormone-positive tumors, whereas many of these have serious side effects. TP10-SRC1_LXXLL_ might be an interesting candidate drug to include in further preclinical investigations.

## Figures and Tables

**Table 1.** Peptide sequences.

Abbreviation	Sequence
TP10	NLS-AGYLLGKINLKALAALAKKIL
R7	NLS-RRRRRRR
TP10-SRC1_LXXLL_	NLS-AGYLLGKINLKALAALAKKIL-YSQTSHK**LVQLL**TTAEQQ
R7-SRC1_LXXLL_	NLS-RRRRRRR-YSQTSHK**LVQLL**TTAEQQ
TP10-SRC1_1222–1245_	NLS-AGYLLGKINLKALAALAKKIL-PQMQQNVFQYPGAGMVPQGEANF
R7-SRC1_1222–1245_	NLS-RRRRRRR-PQMQQNVFQYPGAGMVPQGEANF

NLS, nuclear localization signal PKKKRKV of SV40; TP10, transportan 10; R7, hepta-arginine; TP10-SRC1LXXLL, sequence of SRC1 NR box 1 with *N*-terminal TP10; R7-SRC1LXXLL, sequence of SRC1 NR box 1 with *N*-terminal R7; TP10-SRC1_1222–1245_, randomly chosen non-structured fragment of SRC-1 with *N*-terminal TP10; R7-SRC1_1222–1245_, randomly chosen non-structured fragment of SRC-1 with *N*-terminal R7. LXXLL motif of SRC1 NR box 1 is in bold.
